# Efflux drug transporters at the forefront of antimicrobial resistance

**DOI:** 10.1007/s00249-017-1238-2

**Published:** 2017-07-14

**Authors:** Tahmina Rahman, Benjamin Yarnall, Declan A. Doyle

**Affiliations:** 1 University of Southampton, Biological Sciences, Highfield Campus, Southampton, SO17 1BJ UK; 2 Wessex Kidney Centre, Queen Alexandra Hospital, Cosham, Portsmouth, PO6 3LY UK

**Keywords:** Antimicrobial resistance, Efflux transporters, Persister, RND, Efflux pumps, Antibiotics

## Abstract

Bacterial antibiotic resistance is rapidly becoming a major world health consideration. To combat antibiotics, microorganisms employ their pre-existing defence mechanisms that existed long before man’s discovery of antibiotics. Bacteria utilise levels of protection that range from gene upregulation, mutations, adaptive resistance, and production of resistant phenotypes (persisters) to communal behaviour, as in swarming and the ultimate defence of a biofilm. A major part of all of these responses involves the use of antibiotic efflux transporters. At the single cell level, it is becoming apparent that the use of efflux pumps is the first line of defence against an antibiotic, as these pumps decrease the intracellular level of antibiotic while the cell activates the various other levels of protection. This frontline of defence involves a coordinated network of efflux transporters. In the future, inhibition of this efflux transporter network, as a target for novel antibiotic therapy, will require the isolation and then biochemical/biophysical characterisation of each pump against all known and new antibiotics. This depth of knowledge is required so that we can fully understand and tackle the mechanisms of developing antimicrobial resistance.

## Introduction

Antimicrobial resistance (AMR) describes the ability of microbes to resist the growth inhibitory effects of antimicrobials or antibiotics. The rise of such organisms threatens many of the major advances in modern healthcare as the treatments rely on the implicit ability to allow the body to heal free from a serious infection (Harbarth et al. 2015). AMR has now become a major global threat to public health (Mushtaq 2016). Antimicrobial resistance is present in every country and as a result the United Nations and the World Health Authority have committed to tackling this serious danger to our modern medical approach (WHO 2016). There is also an ever increasing cost to delivering healthcare as a result of AMR. Patients with infections caused by drug-resistant bacteria are at increased risk of poor outcomes or death and consume more healthcare resources than those infected with the same non-resistant strain (Giske et al. 2008; Laxminarayan et al. 2013).

The emergence of resistance is partly due to the natural selection process in microorganisms (Wright and Poinar 2012) but also as a consequence of human activity in our extensive and unregulated use of antibiotics over the last 70 years. The simple outcome of exposing microorganisms to antibiotics forces the bacteria to change to survive. Bacteria adopt a number of molecular mechanisms as they develop resistance to antibiotics. These include (1) mutations that alter the antibiotic target site sufficiently so that it no longer binds; (2) inactivating the antibiotic through enzymatic activity; (3) by-passing the targeted metabolic pathway; (4) over-producing a target protein so that sufficient unbound target can operate normally; (5) decreasing the ability for the antibiotic to be taken up by reducing the number of entry points; (6) actively exporting the drug out of the cell (Schweizer 2012; Nguyen 2016). Clearly, the fastest and simplest mechanism is to pump out the antibiotic using bacterial efflux transporters.

## Efflux transporters

**Fig. 1:**
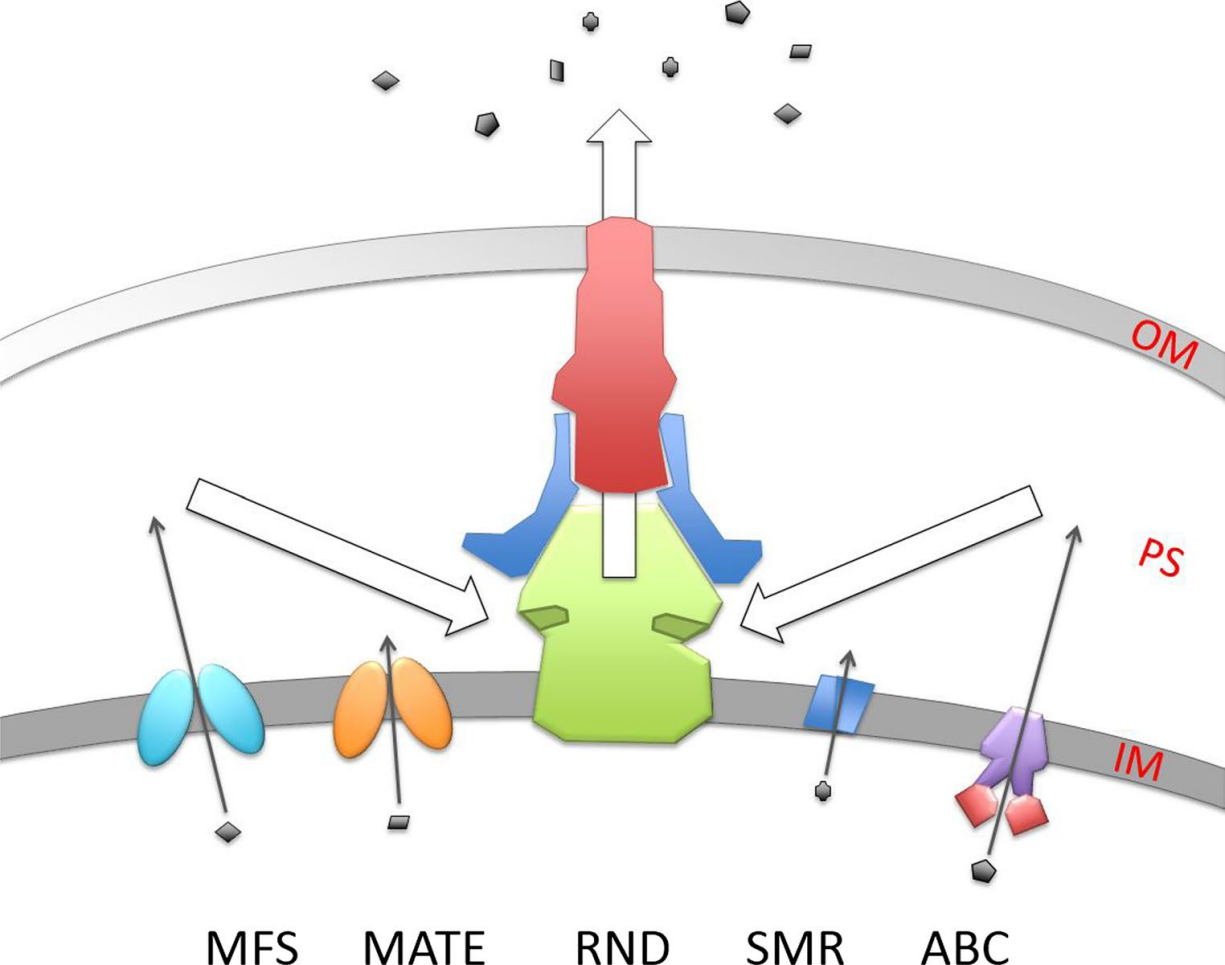
Schematic view of the five antibiotic efflux transporters present in the Gram-negative bacterium *Escherichia coli*. The inner membrane (IM) and outer membrane (OM) separate the periplasmic space (PS). The small *grey shapes* represent a variety of antibiotics and the *arrows* demonstrate the direction of movement of the antibiotics through the transporters to the outside. The RND transporter is part of a tripartite complex consisting of the three subunits: the inner membrane pump, e.g. *E. coli*‘s acrB (*green*), and the outer membrane β-barrel pore, e.g. *E. coli* tolC (*red*), which are linked together by a periplasmic localised protein, e.g. *E. coli arcA* (*blue*). The ABC transporters consist of a transmembrane body in purple and ATP-binding domains in *red*. MFS and MATE families are single genes that form two domains, which are represented by the *two ovals*. Finally, the SMR families of proteins have to dimerise to form the active transporter

Antibiotic efflux mechanisms have been studied in Gram-negative bacteria and nature has implemented a range of efflux transporters that pump out antibiotics. Antibiotic efflux pumps can be divided into five families: the Small Multidrug Resistance (SMR) family, the Multidrug And Toxic compound Extrusion (MATE) family, the Major Facilitator Superfamily (MFS), the ATP-Binding Cassette (ABC) family and the Resistance-Nodulation-cell Division (RND) family (Fig. 1). The diverse amino acid sequences and varied three-dimensional structures of these transporters indicate that divergent evolution has probably been central in their formation (Paulsen 2003). In this scenario, pre-existing metabolite efflux transporter genes were duplicated and then mutated to alter their substrate specificity towards exogenous antibiotics or even used as a means of self-protection against antibiotics produced by the bacterium itself. A new family of biocide transporters has been identified in *Acinetobacter baumannii* that are structurally unrelated to all of the previously known efflux transporters (Hassan et al. 2013). This opens up the possibility that there may well be other efflux transporters to be discovered.

All of the transporters listed above require the input of energy to move their target antibiotic out of the cell. SMR, MATE, MFS and RND families use the electrochemical gradient across the bacterial cell wall while the ABC family use the energy released from the hydrolysis of ATP. It is important to emphasise that bacteria therefore have to expend energy to remove these toxic compounds out of the cell. Another significant point is that bacteria are able to use several antibiotic efflux pathways (Fig. 1). Therefore, there has been an evolutionary selection pressure to develop multiple pathways by which a bacterium can extrude toxic compounds as part of its normal metabolism and as a survival mechanism. These toxic compounds can range from internally produced harmful metabolites, environmentally derived heavy metals, and antibacterial compounds produced from competitors to man-made antimicrobials. The range of toxins that these transporters are actually able to deal with is much larger and includes harmful biocides including harsh chemicals such as disinfectants, herbicides and pesticides (Allen et al. 2006; Nikaido et al. 2011; Staub et al. 2012).

## Endogenous functions of efflux transporters

Understandably, much of the research has focused on the efflux of antibiotics, however, these transporters have existed long before our use of chemicals to control or eradicate bacteria, fungi, weeds or pests. The RND superfamily of efflux proteins was first described as a related group of membrane transport proteins involved in heavy metal resistance (*Ralstonia metallidurans*), nodulation (*Mesorhizobium loti*) and cell division (*E. coli*). These toxic elements are often present in their habitat hence the need for an efflux system (Rademacher and Masepohl 2012; Choudhary and Sar 2016). The RND pump proteins have one member that has a surprising substrate—unfolded polypeptides. This membrane protein in *E. coli* is secDF and forms part of the translocon complex required to move proteins across the bacterial inner membrane. In this case, secDF pulls the translocating polypeptide into the periplasmic space. Even though the substrates are very diverse, both the secDF pulling mechanism and the arcB drug extruding mechanism move their targets in the same direction and use the proton motive force as their energy source (Tsukazaki et al. 2011).

Efflux pumps play a key role in the elimination of organic pollutants and protect the organism from the toxic effects of these pollutants. For example, in *Pseudomonas putida,* a strain resistant to organic solvents such as toluene, increased activity of its RND tripartite efflux pump TtgABC resulted in the extrusion of toluene (Duque et al. 2001). The related efflux transporter EmhABC, from *Pseudomonas fluorescens cLP6a,* extrudes fatty acids after membrane damage or during the natural turnover of the lipid components (Adebusuyi and Foght 2011).

NephAB is another efflux transporter from *Arthrobacter nicotinovorans*, which extrudes methylamine, the end result of nicotine biodegradation within the organism. This membrane protein is an example of the SMR family of drug transporters (Ganas et al. 2007).

In pathogenic bacteria, some efflux pumps are able to extrude agents, such as organic solvents, that are not normally present in the host. This is achieved by upregulating the same antibiotic efflux pumps. For example, the development of *P. aeruginosa* strains which are resistant to organic solvents, is due to the expression of antibiotic efflux pumps (Li and Poole 1999). Hence, not only antibiotics but also organic solvents can generate resistant strains through expression of drug efflux pumps in non-clinical environments. This indicates the importance of the exposure to toxic chemicals present in the environment in the development of antimicrobial resistance.

As well as being a multidrug efflux transporter, the *E. coli* MFS mdfA pump has been shown to be important for growth at alkaline pH values. This is believed to involve the extrusion of K^+^ in exchange for protons to maintain the internal pH with high external pH values (Lewinson et al. 2004). *E. coli* utilises another MFS transporter, mdtM (Paul et al. 2014), to lower the concentrations of bile salts while *Campylobacter jejuni* uses its cmeABC RND transporter to achieve the same result (Lin et al. 2005). As well as extruding compounds that come from outside the cell, e.g. antimicrobials from competitors, toxic heavy metals and organic compounds, drug efflux transporters have a role in eliminating potentially toxic compounds produced as a consequence of cellular metabolism. Hence, the antibiotic efflux transporters form only part of an overall detoxifying system involving a large range of coordinated membrane proteins.

## Adaptive resistance involves efflux transporters

Antibiotics have an optimum killing zone concentration. Concentrations far below this level will have no effect on bacterial growth. These lower levels are, however, not undetected by the bacteria. Bacteria are able to respond to this low antibiotic level and prepare themselves by upregulating efflux transporters among other activities. This results in a stronger defence mechanism that allows bacteria to become resistant to higher concentrations of antibiotics that would normally kill them. As part of this adaptive resistance mechanism, the resistance is reversible when the antibiotic is removed (Viveiros et al. 2002; Sandegren 2014; Motta et al. 2015). It is well known that mutations can alter the expression of drug efflux transporters (Chen et al. 2007; Brzoska et al. 2013; Curiao et al. 2016) but epigenetics plays an important role in this reversible mechanism (Adam et al. 2008; Motta et al. 2015). Perhaps it is not unexpected that various pathways are upregulated upon exposure to antibiotics as most antibiotics are derived from natural microbial products.

Even though bacteria possess a range of efflux transporters, dealing with an antibiotic attack appears to have a cost associated with it as the organism has to spend additional resources and energy to defend itself. Once the threat has dispersed, the levels of efflux transporters are reduced, allowing valuable metabolic resources to be re-directed potentially back towards growth and division.

## Efflux transporters and persisters

Phenotypic changes to bacteria also play a major role in antibiotic resistance. Within any population of bacteria, there is a small but important number of phenotypic variations that are resistant to the toxic effect of antibiotics. Organisms with an altered phenotype are believed to exist as a mechanism to deal with potentially catastrophic conditions. For example, in the case of antibiotic exposure, a high dose of a particular antibiotic could kill almost an entire colony of bacteria, however, there are always a few cells that are able to withstand the harsh conditions. These are called persisters. These altered bacteria have been shown to be quiescent and do not grow or divide even under conditions that normally favour cell growth (Balaban et al. 2004). These persister cells have not been genetically modified as, once the threat has dissipated, the new progeny are as susceptible to antibiotics as the original parent cells (Keren et al. 2004; Maisonneuve et al. 2013).

For cells to remain in a dormant phase even when there are sufficient nutrients available for growth, an alteration in metabolism must have occurred. This strategy is a key feature of persister cells (Li and Zhang 2007; Ma et al. 2010; Amato et al. 2014). This has been exploited as a means of killing persister cells by using chemicals that can encourage them to become more metabolically active thus allowing particular antibiotics to be effective (Allison et al. 2011; Barraud et al. 2013; Thorsing et al. 2015).

Dormancy in persister cells does not mean total metabolic inactivity. Persister cells still have to sample their environment and respond suitably. As many antibiotics are hydrophobic in character they can cross membranes without requiring a proteinaceous pathway. This could result in a build-up of dangerous antibiotic levels for the bacterium. Indeed, the cells are still partially metabolically active and may produce toxic compounds that also need to be expelled. In such cases, it would be expected that the bacterial efflux systems are upregulated. This has recently been demonstrated in *E. coli* persister cells in which Pu et al. (2016) demonstrate that the antibiotic efflux systems, particularly those involving tolC, have enhanced efflux activity with lower intracellular concentrations of antibiotics.

## Biofilms and efflux transporters

Persister cells are known to reside as a small proportion of the general bacterial population, however, their numbers are increased in the protective environment of the biofilm. A biofilm is formed when free-swimming microorganisms attach to a surface and build a protective barrier made of polymers such as DNA and protein but mainly polysaccharides. These biofilms are a heterogeneous mix of microorganisms and, due to a biofilm’s physical structure, the biofilm can possess at least two general environments that gradually transform from one to the other as a dynamic collective unit of bacteria. They are (1) an inner oxygen- and nutrient-deprived region that contains dormant cells and (2) an aerobic outer layer that contains metabolically active cells. As such there will be a varied population of phenotypic and genotypic microorganisms that make up any mature biofilm (Stewart and Franklin 2008). The physical barrier that a biofilm offers is a major part of this defensive strategy. The protective barrier can block entry of antibiotics as well as immune cells. It also protects against physical movement of microorganisms in dynamic fluids and can hold onto moisture and thus protect against dehydration. The disadvantage for a microorganism is being walled into an environment with limited resources and plenty of close competition. However, the advantages outweigh the disadvantages particularly when the microorganisms are subjected to antimicrobial attack with antibiotics.

Predictably, the microorganisms within biofilms combine a number of the already mentioned antimicrobial resistance strategies to protect themselves. They tend to be slow growing (Lewis 2007) with altered metabolic states (Amato et al. 2014). High doses of antibiotics can kill almost all of the cells within a biofilm but a few remain to recolonise the environment. These cells that can recolonise are persister cells (Brooun et al. 2000). It has been found that there are more persister cells in a biofilm than in free-floating bacteria in planktonic cultures (Singh et al. 2009; Yang et al. 2015). Another parallel between planktonic and biofilm localised persisters is the requirement for efflux pumps (Kvist et al. 2008; Matsumura et al. 2011; Liao et al. 2013). Apart from the concept that biofilm persisters can recolonise their environment in a similar fashion to the planktonic version, it is not clear how, or if, the biofilm persisters contribute to the formation and/or maintenance of the biofilm’s structure.

## Efflux transporters as the first line of defense against antibiotics

Throughout the hundreds of millions of years of microbial existence, the exposure of microorganisms to potentially lethal chemicals such as antibiotics has, through evolution, resulted in a series of changes aimed at combating such threats. These alterations include physically moving away (Tso and Adler 1974), genetic and phenotypic changes, adopting a communal lifestyle in biofilms and even as part of swarming (Lai et al. 2009; Butler et al. 2010; Harshey and Partridge 2015). However, at a single cellular level, it now appears that the front line of their defense is their drug efflux transporters.

It has been observed that, for many clinical isolates of antibiotic-resistant bacteria, there is an upregulation of efflux transporters. If we hypothesise that a transporter is central to the resistant phenotype, then deleting or disabling the transporter should result in a more sensitive antibiotic strain. However, this was not observed when one of these transporters was disabled; the level of antibiotic resistance remained almost unchanged (Yasufuku et al. 2011; Costa et al. 2011; Kosmidis et al. 2012; Adabi et al. 2015). The problem with this approach when determining transporter function is that it does not take into account redundancy in the system. As mentioned above, bacteria possess not just one family of structurally related transporters but several drug efflux transporter families (Fig. 1). Therefore, the lack of effect on antibiotic sensitivity after deletion of one transporter could be explained by the fact that more than one transporter is able to efflux the same antibiotic. This has been demonstrated in *E. coli* (Tal and Schuldiner 2009; Paul et al. 2014; Wang et al. 2015). Three transporters from three families were able to compensate for the loss of another efflux transporter. Also, in the same way that the RND CusCFBA copper efflux system in *E. coli* pumps copper ions out of the periplasmic space (Delmar et al. 2014), the AcrAB-TolC RND efflux pump appears to function as a general pump for the periplasmic space, i.e. it pumps out the majority of antibiotics that are present in the periplasmic space. The single component efflux pumps from the MFS, SMR and MATE families, as well as the ABC antibiotic efflux transporters, move antibiotics from the cytoplasm to the periplasmic space. The antibiotics are then moved outside the organism by the RND pump complex with the linker protein to the porin-like TolC channel (Fig. 1; Tal and Schuldiner 2009).

Taking this a step further, this network of efflux transporters is not only helping to protect against antimicrobials but is in fact the first line of defense of bacteria against a toxic chemical attack (Shuster et al. 2016). Without efflux transporters, the quickest bacterial response would be to switch on the production of genes that actively destroy or disable the chemical threat. This adaptive process takes time to achieve, potentially too long for survival. Longer term antimicrobial adaptations, like mutations, are likely to require too much time as would the relatively lengthy process of forming the fortress of a biofilm. In this process the network of efflux transporters plays a vital role. These constitutively expressed membrane proteins ensure that the internal concentration of antibiotic is kept at a sufficiently low level to allow the bacteria to adopt longer term protective measures against the antibiotic such as changes in expression levels of the appropriate genes and/or mutations. In such a scenario, you would expect an increase in the number of efflux pumps as the initial defensive step to augment the activity of those already present. This has been observed in the case of *Mycobacterium avium*-*M* when treated with azithromycin (Schmalstieg et al. 2012) and *Helicobacter pylori* eradication with metronidazole as part of triple therapy (Tsugawa et al. 2011), implying that the first line of defense against antibiotics for other bacteria is the efflux transporter network.

## Functionally characterising antibiotic transporters

One of the standard methods of characterising drug efflux transporters is by recording minimal inhibitory concentrations in the presence and absence of the transporter in vivo. This has proved to be very successful and provided extensive information for many transporters (Edgar and Bibi 1997; Sulavik et al. 2001; Nishino and Yamaguchi 2001). Such evidence from whole-cell experiments do provide excellent results in many instances but can leave the experiments open to incorrect interpretation as the gene of interest may be indirectly enhancing resistance. Genomics and next-generation sequencing technologies are providing enormous amounts of data on the genes of efflux transporters but the substrates for these membrane protein transporters should be properly identified rather than relying on bioinformatic identification. Therefore, as for all enzymes, biochemical and biophysical characterisation of transporters should involve isolating the transporter and functionally characterising it in a controlled environment (Blair and Piddock 2016). Extensive efflux characterisation will be required if we are going to fully understand how this overlapping network of transporters operates and therefore how resistance mechanisms can be tackled as part of an antibiotic drug development programme.
